# Functionalized Phenyl Methanaminium Salts Provide Highly Stable Perovskite Solar Cells

**DOI:** 10.1021/acsami.5c00985

**Published:** 2025-03-17

**Authors:** Mustafa Yaşa, Esra Bag Celik, Xiao-Xin Gao, Zeynep Gözükara Karabağ, Ummugulsum Gunes, Olga A. Syzgantseva, Maria A. Syzgantseva, Liping Zhong, Andreas Züttel, Paul J. Dyson, Mohammad Khaja Nazeeruddin, Levent Toppare, Selcuk Yerci, Gorkem Gunbas

**Affiliations:** 1 ODTÜ-GÜNAM, Middle East Technical University, Ankara 06800, Türkiye; 2 Department of Polymer Science and Technology, Middle East Technical University, Ankara 06800, Türkiye; 3 Institute of Chemical Sciences and Engineering, École Polytechnique Fédérale de Lausanne, Lausanne 1015, Switzerland; 4 Department of Chemistry, Middle East Technical University, Ankara 06800, Türkiye; 5 Department of Chemistry, Lomonosov Moscow State University, Moscow 119991, Russia; 6 Department of Physics, Mendeleev University of Chemical Technology, Moscow 125047, Russia; 7 Department of Micro and Nanotechnology, Middle East Technical University, Ankara 06800, Türkiye; 8 Department of Electrical-Electronic Engineering, Middle East Technical University, Ankara 06800, Türkiye

**Keywords:** photovoltaics, perovskite solar cells, phenyl methanaminium salt, passivation, recombination states

## Abstract

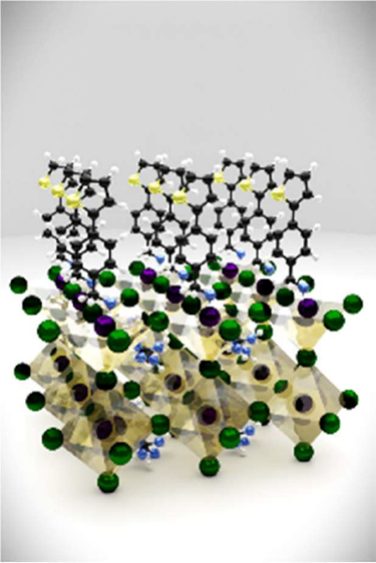

One of the leading approaches to enhancing the performance and stability of perovskite solar cells (PSCs) involves passivating the perovskite surface and grain boundaries with large ammonium salts. Here, we report the synthesis of furan-, thiophene-, and selenophene-functionalized phenyl methanaminium iodide salts (**FPMAI**, **TPMAI**, and **SPMAI**) and their application as passivating agents on 3D [(Cs_0.04_FA_0.85_MA_0.11_)Pb(I_0.96_Br_0.01_Cl_0.03_)_3_] perovskite. The **TPMAI**-passivated PSCs performed the best and achieved a power conversion efficiency (PCE) of 23.15% compared to the reference (without a passivating agent) at 20.91%. Efficiencies reduced to 98 and 54% of the initial value after 1250 h of continuous illumination for **TPMAI**-treated PSCs and the reference, respectively. DFT calculations revealed that **TPMAI** offers superior passivation, disfavoring iodine vacancy formation. Our findings highlight the potential of functionalized PMAI salts as passivation agents for improved efficiency and stability in PSCs.

## Introduction

Metal-halide PSCs became the leading competitor to conventional silicon-based solar cells due to their outstanding optoelectronic properties, low fabrication cost, and ease of fabrication.^1−4^ Miyasaka et al. reported the first PSC, demonstrating an efficiency of 3.8%,^5^ and since this seminal work, extensive research has been conducted and now the efficiency of PSCs has reached to 27%.^6^ Despite the remarkable efficiencies of PSCs, fabricating highly stable perovskite solar cells remains challenging due to their instability due to external and internal factors.^7−9^

One of the significant challenges of using perovskite films in solar cells is the formation of the defect states on their surfaces and/or in the grain boundaries, which can lead to reduced efficiency and long-term instability.^10−13^ It is known that the recombination in trap states either on the surface of the perovskite absorber layer or in the bulk strongly impacts the open-circuit voltage (*V*_OC_) of PSCs.^14−17^ In general, the surface of the perovskite is highly prone to defect formation and exhibits a high defect density and contributes to charge recombination and short carrier diffusion lengths.^18^ In addition, interfacial iodine vacancies affect both the efficiency and stability of PSCs. The vacancies are typically present at grain boundaries and the interface between the perovskite and adjacent layers (electron transport layer (ETL) and hole transport layer (HTL)) and create localized defect states that can trap charge carriers, subsequently leading to increased recombination losses and reduced charge transport. Therefore, passivation of the surface defects on perovskite films helps to suppress ion migration^13,14,19^ and reduce recombination occurring at the interface between the active layer and the HTL.^20,21^

Among the various compounds used for surface passivation, ammonium halides show excellent properties, suppressing nonradiative recombination, either by forming a low dimensional (2D) perovskite layer or only acting as a passivation layer.^22−29^ Phenyl alkyl ammonium salts have been shown to improve the performance of PSCs by reducing surface defects,^21^ creating an efficient and stable planar structure, and scavenging deep-level.

**Figure 1:**
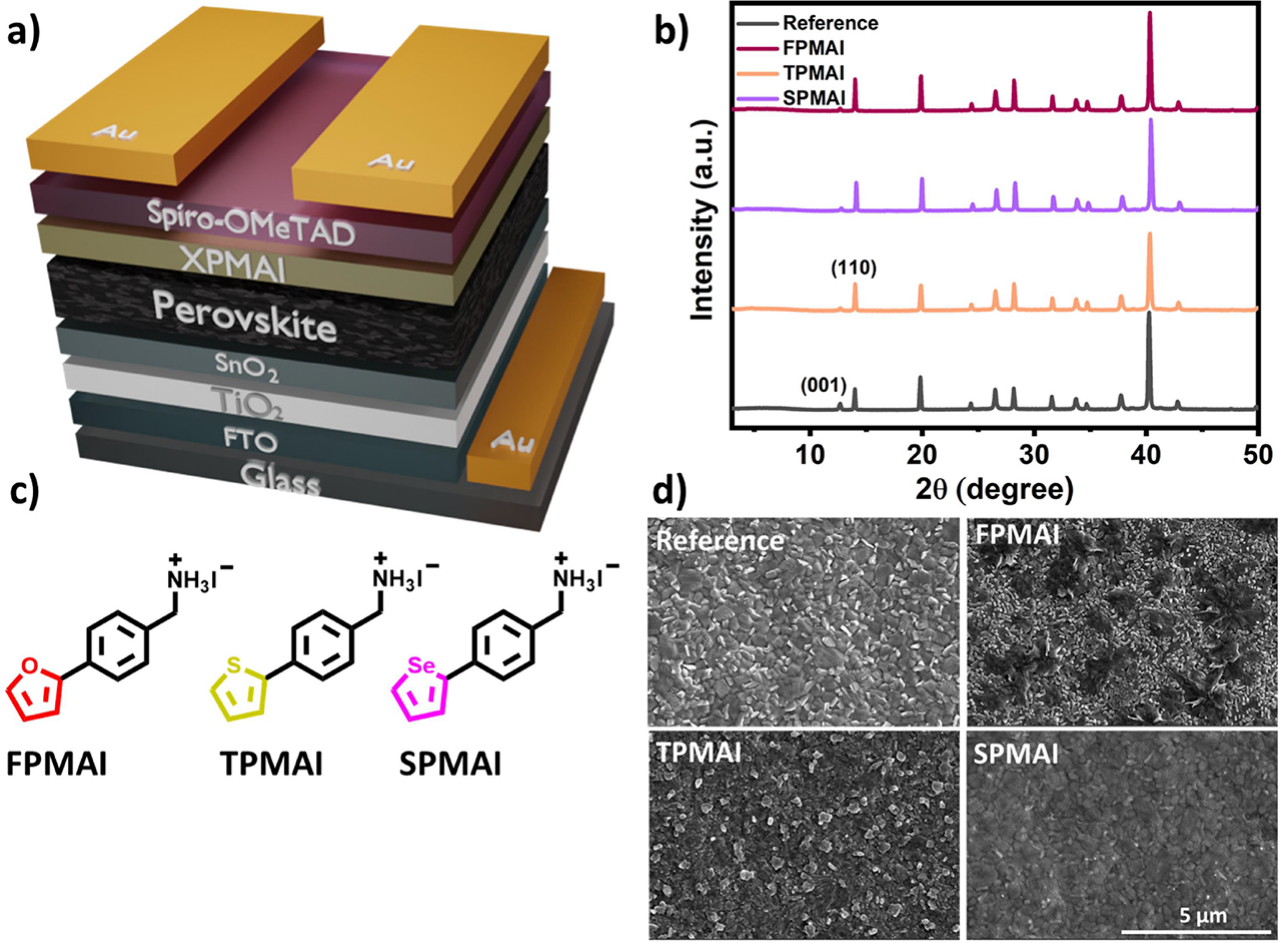
(a) Schematic of the device structure; (b) X-ray diffraction (XRD) patterns of reference perovskite and **FPMAI**-, **TPMAI**-, and **SPMAI**-treated perovskite films. (c) Structures of **FPMAI**, **TPMAI**, and **SPMAI** and (d) top-view SEM images of reference, **FPMAI**-, **TPMAI**-, and **SPMAI**-treated perovskite films.

Herein, we describe three PMAI-based ammonium salts, namely, (4-(furan-2-yl)phenyl) methanaminium iodide (**FPMAI**), (4-(thiophen-2-yl)phenyl) methanaminium iodide (**TPMAI**), and (4-(selenophen-2-yl)phenyl) methanaminium iodide (**SPMAI**) (Figure 1c). These salts could effectively decrease the bulk iodine vacancy content and thus passivate the recombination states, improving the perovskite stability and enhancing the performance due to the limitation of nonradiative recombination losses and improved charge transport across the interface.^30−32^ The salts were used as passivating agents in n–i–p-type PSCs comprising a CsFAMA-based triple-cation perovskite. The resulting PSCs exhibit enhanced *V*_OC_ and PCE and significantly increased stability compared to those of the reference device.

## Results and Discussion

The chalcogenophene-functionalized PMAI salts, FPMAI, **TPMAI**, and **SPMAI**, were prepared via coupling reactions under mild reaction conditions and in good yields (full characterization is provided in the Supporting Information). Using these PMAI salts as passivation agents, PSCs were fabricated in the n–i–p device architecture with a glass/FTO/*c*-TiO_2_/*m*-TiO_2_/SnO_2_/[(Cs_0.04_FA_0.85_MA_0.11_)Pb(I_0.96_Br_0.01_Cl_0.03_)_3_]/XPMAI-based salts/2,2′,7,7′-tetrakis[*N*,*N*-di(4-methoxyphenyl)amino]-9,9′-spirobifluorene (Spiro-OMeTAD)/Au configuration (Figure 1a).

XRD analysis was performed to observe the influence of the PMAI salts on the crystallinity of the 3D perovskite films. The PMAI-treated films do not contain a peak (typically below 10°)^25^ corresponding to the formation of a 2D perovskite layer.^33^ Indeed, the XRD patterns of the films are very similar (Figure 1b), indicating that PMAI treatment does not alter the crystallinity of the films to a great extent. Unconverted PbI_2_ and 3D perovskite peaks are observed at 12.7 and 14.0°, respectively, for the reference and the PMAI-treated films. However, the peak observed corresponding to PbI_2_ has a lower intensity in the PMAI-treated films, presumably due to disaggregation of excess PbI_2_ on the perovskite surface, rather than forming a 2D perovskite layer.^34^

Scanning electron microscopy (SEM) images (Figure 1d) show that PMAI treatment changes the surface morphology of the films. The **TPMAI**- and **SPMAI**-treated films show more homogeneous film surfaces compared to the **FPMAI**-treated film where local aggregation of the salt was observed. In addition, excess PbI_2_ (the bright spots in the SEM images) on the reference film is considerably reduced upon treatment with the PMAI salts, consistent with the XRD patterns.

**Figure 2:**
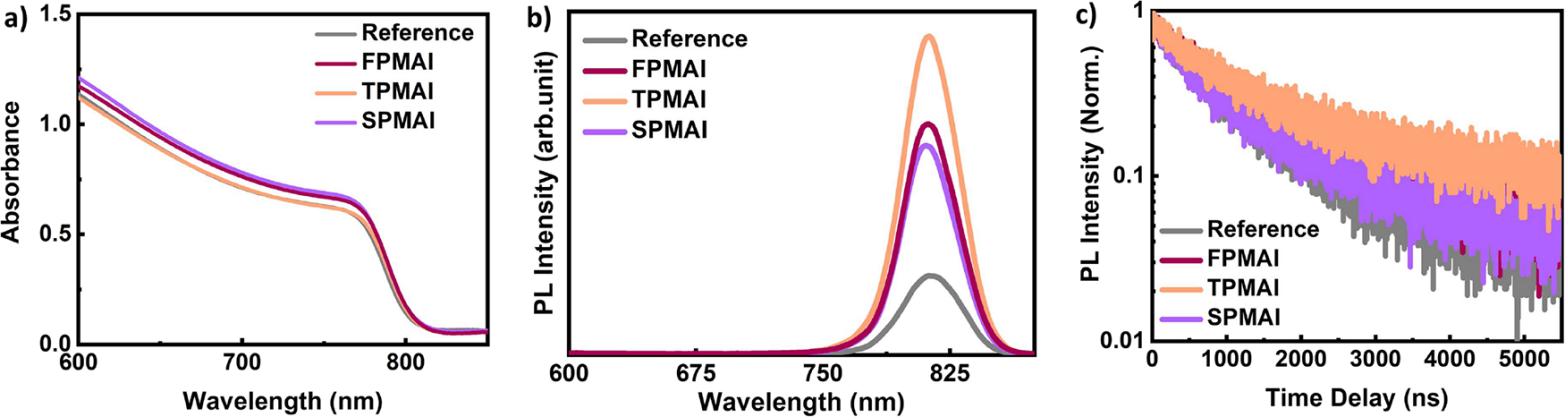
(a) UV–vis absorption spectra of the reference and **FPMAI**-, **TPMAI**-, and **SPMAI**-treated films. (b) PL spectra of the reference, **FPMAI**, **TPMAI**, and **SPMAI** films; (c) TRPL spectra of the reference, **FPMAI**, **TPMAI**, and **SPMAI** films.

As depicted in the UV–vis absorption spectra, see Figure 2a, the PMAI salts, located at the rear side of the 3D perovskite, do not contribute to the absorption of the perovskite film.^23^ An increase in the PL intensities (Figure 2b) is observed for all the PMAI-treated films, which should lead to a decrease in nonradiative recombination due to passivation of defects found in the perovskite or on its surface.^26^ Among the **FPMAI**, **TPMAI**, and **SPMAI**-treated films, **TPMAI** shows the highest PL intensity and the longest PL decay time (Figure 2c). The ionization energies of the PMAI salts and perovskite layer were determined using ultraviolet photoelectron spectroscopy (UPS) as −6.10, −5.51, −6.03, and −5.90 eV for **FPMAI**, **TPMAI**, **SPMAI**, and the perovskite layer, respectively. The valence band maximum cutoff regions and work function regions are provided in Figure S1. The highest occupied molecular orbital (HOMO) energy in the salt-passivated films is lower than that of the reference film, leading to enhanced performance although there is a greater mismatch with the HTL.^25^

**Figure 3:**
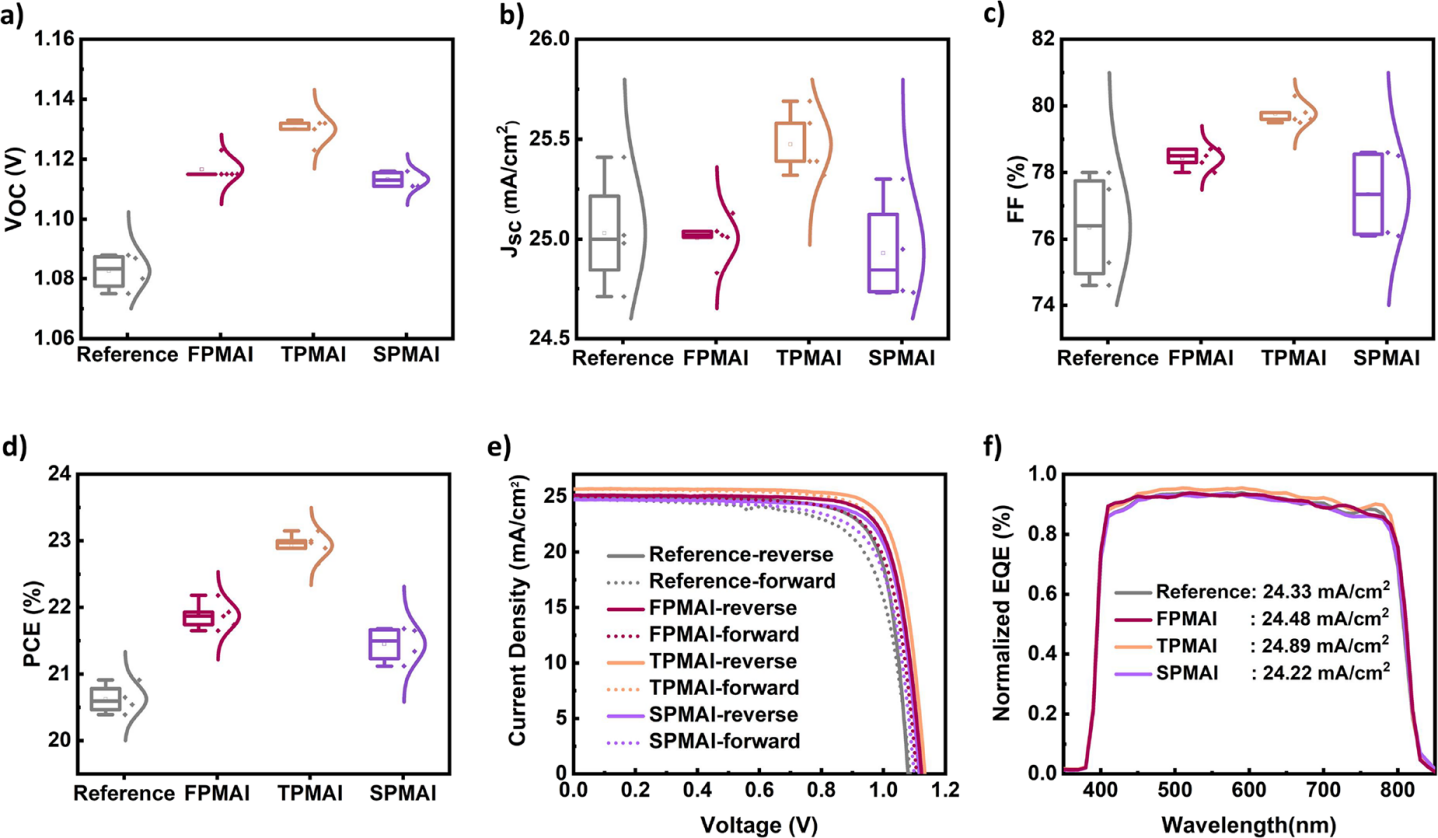
Photovoltaic statistics of reference and XPMAI-treated perovskite solar cells. (a) *V*_OC_, (b) *J*_SC_, (c) FF, (d) PCE, and (e) *J–V* curves of the reference and XPMAI-treated champion devices recorded under one-sun illumination. (f) External quantum efficiency (EQE) spectra of reference and XPMAI-treated champion devices and calculated photocurrent values.

Figure 3a–d shows the average *V*_OC_, short-circuit current density (*J*_SC_), fill factor (FF), and PCE of PSCs containing perovskite films treated with **FPMAI**, **TPMAI**, **SPMAI**, and the reference. The average *V*_OC_ is 1.08 V for the reference device, whereas it is 1.11, 1.13, and 1.12 V for the **FPMAI**, **TPMAI**, and **SPMAI** devices, respectively, consistent with the enhanced PL intensity and PL lifetimes (Figure 2b,c). The average *J*_SC_ of the PMAI-treated devices and the reference devices are similar, which aligns with the absorption spectra (Figure 2a). The reference device has an average PCE of 20.63% compared to 21.87% for **FPMAI**, 22.93% for **TPMAI**, and 21.45% for **SPMAI**.

Figure 3e shows the *J*–*V* curves of the champion PSCs. The highest efficiency of the reference device is 20.91%, whereas the highest efficiency is 22.18% for **FPMAI**, 23.15% for **TPMAI**, and 21.65% for **SPMAI** devices. The champion device (**TPMAI**, 23.15%) exhibited a *V*_OC_ of 1.13 V, a *J*_SC_ of 25.67 mA/cm^2^, and an FF of 80.7%.

The *J*_SC_ values obtained by integrating the EQE spectra (Figure 3f) are in good agreement with those directly measured. Further measurements for the hysteresis indices (HI) revealed 7.75, 4.69, 7.20, and 4.78% for the reference, **TPMAI**, **SPMAI**, and **FPMAI** devices, respectively (Table S1). The reduced HI of the passivated films may be attributed to retarded ion migration.^35^

**Figure 4:**
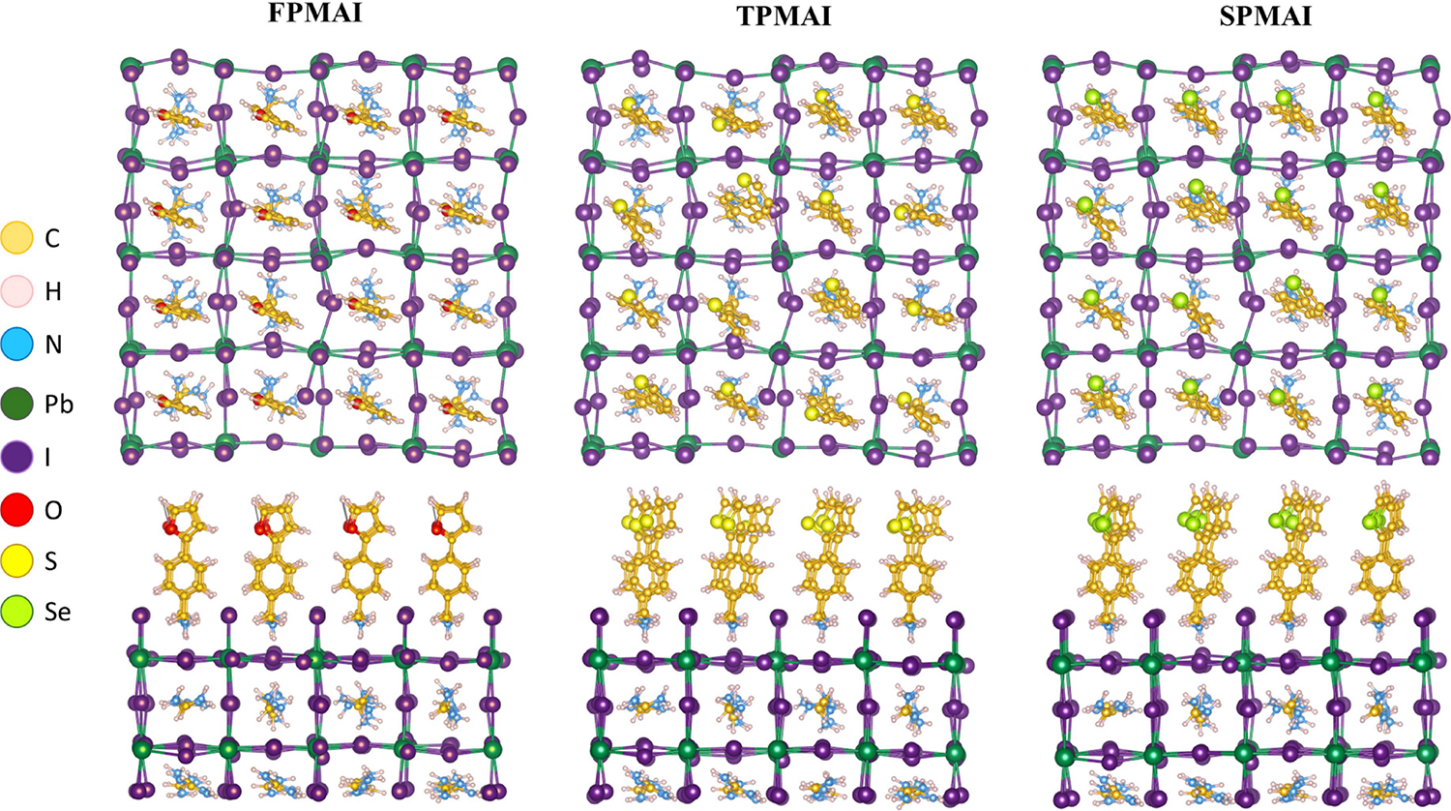
Passivation of the perovskite layer via vertical adsorption of **FPMAI**, **TPMAI**, and **SPMAI** modeled by DFT.

Modeling of the adsorption of the three different PMAI salts as monolayers on a 3D perovskite surface was performed using density functional theory (DFT), see Figure 4. The adsorption energy of the three salts is similar for **FPMAI** (−1.19 eV/molecule), **TPMAI** (−1.21 eV/molecule), and **SPMAI** (−1.20 eV/molecule) (Table 1). When the salts are stacked, **TPMA** cations form S–S attractions that further enhance the stability of the monolayer and related interactions are not observed for the other salts.

**Table 1:** Calculated Vertical Adsorption Energies of the PMAI Salts on the FAPbI_3_ Surface and Calculated Formation Energies of Iodine and Interfacial Iodine Vacancies in the XPMAI-Passivated FAPbI_3_ Perovskites

compound	adsorption energies (*E*_ads_(XPMAI-ML), eV)	formation energies of iodine vacancies (Δ_f_*E* (*I*_vac_), eV)	formation energies of interfacial iodine vacancies (Δ_f_*E* (*I*_vac_), eV)
FPMAI	–1.19	0.30	0.03
TPMAI	–1.21	0.34	–0.33
SPMAI	–1.20	0.05	–0.19

The formation energies of iodine vacancies (Δ_f_*E* (*I*_vac_)) within the passivated perovskite samples (Table 1) were also estimated using DFT, with the highest formation energy of iodine vacancy, for the **TPMAI**-passivated perovskite, with a Δ_f_*E* (*I*_vac_) value of 0.34 eV. Therefore, **TPMAI** should have the lowest iodine vacancy *I*_vac_ concentration. Note that iodine vacancies are unfavorable for the charge carrier transport and diminish the charge carrier lifetime due to undesirable recombination. Interestingly, among the three samples, the **TPMAI**-passivated perovskite has the lowest energy requirement for the removal of interfacial iodine vacancies near the salt monolayer (Table 1). This suggests that iodine atoms experience the highest driving force to migrate toward the **TPMAI**-perovskite interface, thereby promoting the stoichiometric integrity of bulk FAPbI_3_.

**Figure 5:**
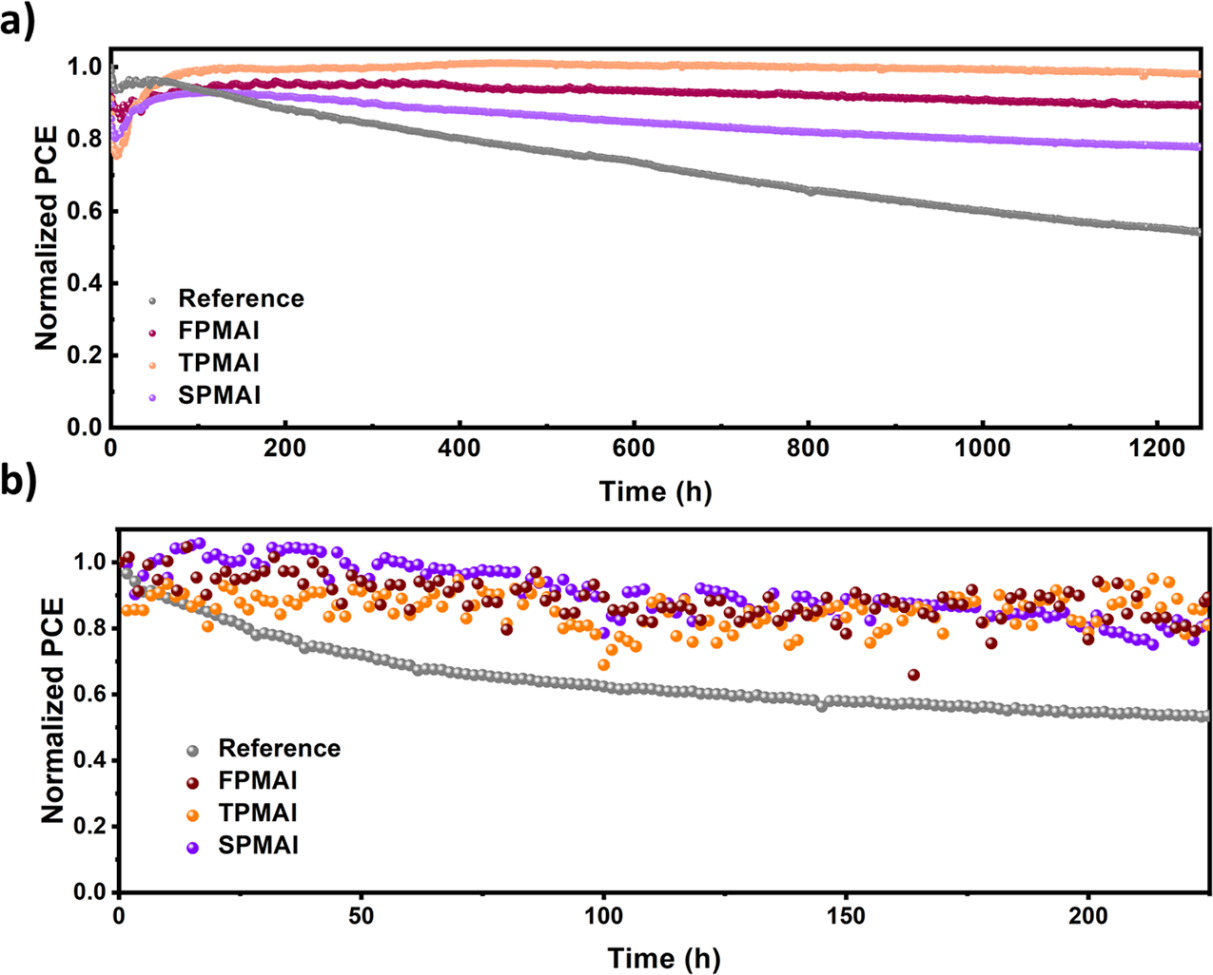
(a) Light-induced stability of the reference and **FPMAI**-, **TPMAI**-, and **SPMAI**-treated PSCs. The devices were measured under N_2_ at room temperature under constant illumination (LED source, ≈1 sun) at a maximum power point for 1250 h and (b) thermal stability of reference and **FPMAI**-, **TPMAI**-, and **SPMAI**-treated cells. The devices were measured under N_2_ at 65 °C.

The stability of the devices under maximum power point (MPP) tracking conditions was conducted for 1250 h in an inert environment (Figure 5a). The reference device maintained 54% of its initial efficiency after 1250 h, whereas **SPMAI**, **FPMAI**, and **TPMAI** retained 78, 89, and 98%, respectively. The trend in stability is consistent with the DFT calculations of iodine vacancy formation energies and highlights the importance of bulk iodine vacancy passivation not only for fine-tuning PSC efficiency but also for improving long-term stability. A thermal stability test was also conducted under N_2_ at 65 °C alongside the MPP tracking stability test. While the reference device lost nearly half of its initial efficiency (54 ± 1%) after 225 h, **SPMAI**, **FPMAI**, and **TPMAI** retained 79 ± 3, 82 ± 12, and 87 ± 7% of their initial performance, respectively (Figure 5b). Additionally, contact angle measurements were conducted (Figure S2) and **TPMAI** has the highest angle (79.3° vs 70.5° for the reference), demonstrating its potential to act as a hydrophobic barrier protecting the perovskite layer from adventitious moisture.

## Conclusions

Furan-, thiophene-, and selenophene-substituted phenyl methanaminium iodide salts (**FPMAI**, **TPMAI**, and **SPMAI**, respectively) were synthesized and applied as passivating agents on 3D [Cs_0.04_FA_0.85_MA_0.11_)Pb(I_0.96_Br_0.01_Cl_0.03_)_3_] perovskite. Notably, **TPMAI** stands out among these salts, as it results in a lower adsorption energy and passivates bulk iodine vacancies, thus favoring stability and performance. A champion PCE of 23.15% was achieved using **TPMAI**, higher than that of the reference device with a champion PCE of 20.91%. Long-term stability studies revealed that a **TPMAI**-treated device retained 98% of its initial efficiency after 1250 h of continuous illumination, whereas the reference device retained only 54%. Thermal stability also improved significantly with the treatment of TPMAI (87% vs 54%). Compared with other salts, **TPMAI** forms intermolecular interactions through the formation of S–S bonds that substantially improve the stability of the perovskite structure while also reducing iodine vacancy formation.

## Experimental Section

### Material Synthesis

#### Tributyl(thiophen-2-yl)stannane^36^

Under an Ar atmosphere, to a 100 mL flask charged with a magnetic stir bar was introduced thiophene (3 g, 35.6 mmol). Dry tetrahydrofuran (THF) (60 mL) was added, and the solution was stirred for 5 m. The temperature of the solution was cooled to −78 °C. *n*-Butyllithium solution (*n*-BuLi) (16 mL, 2.5 M in hexane) was added dropwise, and the reaction mixture was allowed to stir for 1 h at the same temperature. Tributyltin chloride (SnBu_3_Cl) (10.7 mL, 40 mmol) was added slowly, and the reaction mixture was warmed to the ambient temperature. The reaction mixture was stirred overnight, and the solvent was removed. The crude product was diluted with dichloromethane (DCM), and the organic layers were washed with sodium bicarbonate (NaHCO_3_), water, and brine. Combined organic layers were dried over magnesium sulfate (MgSO_4_), and the solvent was removed. A yellow oil was obtained and used without further purification. Yield: 12.45 g, 93%.

^1^H NMR (400 MHz, CDCl_3_): δ 7.70 (tt, *J* = 5.2, 3.0 Hz, 1H), 7.33 (dt, *J* = 4.8, 2.9 Hz, 1H), 7.29–7.23 (m, 1H), 1.76–1.48 (m, 8H), 1.48–1.30 (m, 8H), 1.29–1.06 (m, 7H), 0.96 (tt, *J* = 7.2, 2.0 Hz, 9H). ^13^C NMR (100 MHz, CDCl_3_): δ 136.0, 135.0, 130.4, 127.7, 28.8, 27.1, 13.5, 10.7.

#### 4-(Thiophen-2-yl)benzonitrile^37^

Under an Ar atmosphere, 4-bromobenzonitrile (0.5 g, 2.75 mmol) and bis(triphenylphosphine)palladium(II) dichloride (PPh_3_)_2_PdCl_2_ (40 mg) were dissolved in dry THF (10 mL). Tributyl(thiophen-2-yl)stannane (1.25 g, 3.3 mmol) was added. The reaction mixture was heated to 65 °C and stirred for 16 h under reflux. Following cooling to ambient temperature, the mixture was extracted with DCM, and the collected organic layers were washed with brine and dried over MgSO_4_. The solvent was removed, and the crude product was purified on silica gel (SiO_2_, hexane:DCM, 1:1) to afford a white powder. Yield: 0.38 g, 75%. ^1^H NMR (400 MHz, CDCl_3_): δ 7.79–7.58 (m, 4H), 7.50–7.40 (m, 2H), 7.40 (s, 1H), 7.12 (ddd, *J* = 8.9, 5.1, 3.6 Hz, 1H). ^13^C NMR (100 MHz, CDCl_3_): δ 141.9, 138.5, 132.6, 128.4, 126.9, 125.9, 125.0, 118.7, 110.4.

#### 4-(Selenophen-2-yl)benzonitrile^37^

4-Bromobenzonitrile (0.5 g, 2.75 mmol) and (PPh_3_)_2_PdCl_2_ (40 mg) were dissolved in dry THF (10 mL) under an Ar atmosphere. Tributyl(selenophen-2- yl)stannane (1.4 g, 3.3 mmol) was added slowly. The reaction mixture was heated to 65 °C and allowed to stir for 16 h under reflux. The reaction was cooled to ambient temperature, and the mixture was extracted with DCM. The collected organic layers were washed with brine and dried over MgSO_4_. The solvent was removed under reduced pressure, and the crude product was purified on silica gel (SiO_2_, hexane:DCM, 1:1). Yield: 0.6 g, 93%. ^1^H NMR (400 MHz, CDCl_3_): δ 8.08 (dd, *J* = 5.5, 1.1 Hz, 2H), 7.64 (s, 3H), 7.59 (dd, *J* = 3.9, 1.1 Hz, 1H), 7.37 (dd, *J* = 5.6, 3.9 Hz, 1H). ^13^C NMR (100 MHz, CDCl_3_): δ 148.1, 140.5, 132.6, 132.4, 130.9, 127.3, 126.4, 118.7, 110.4.

#### 4-(Furan-2-yl)benzonitrile^37^

Under an Ar atmosphere, in a 50 mL Schlenk tube, 4-bromobenzonitrile (0.43 g, 2.33 mmol) and tetrakis(triphenylphosphine)palladium(0) Pd(PPh_3_)_4_ (0.19 mg) were dissolved in dry toluene (20 mL). The mixture was heated to 50 °C, and tributyl(furan-2-yl)stannane (1.0 g, 2.8 mmol) was added slowly. The reaction mixture was heated to reflux and stirred overnight. Following cooling to ambient temperature, the solvent was removed and the crude product was eluted through silica gel (SiO_2_, hexane:DCM, 1:1). Yield: 0.37 g, 94%. ^1^H NMR (400 MHz, CDCl_3_): δ 7.74 (dt, *J* = 8.4, 1.9 Hz, 2H), 7.66 (dt, *J* = 8.4, 1.9 Hz, 2H), 7.54 (q, *J* = 2.1 Hz, 1H), 6.82 (dt, *J* = 3.7, 2.2 Hz, 1H), 6.53 (dq, *J* = 3.5, 2.0 Hz, 1H). ^13^C NMR (100 MHz, CDCl_3_): δ 150.9, 143.5, 134.5, 132.4, 123.8, 118.8, 112.1, 110.1, 108.0.

#### (4-(Thiophen-2-yl)phenyl)methanamine^38^

Under an Ar atmosphere, 4-(thiophen-2-yl)benzonitrile (0.34 g, 1.48 mmol) was dissolved in dry THF (10 mL). Lithium aluminum hydride (LiAlH_4_) (0.21 g, 5.5 mmol) was added slowly. The reaction mixture was heated to 50 °C, and the mixture was allowed to stir overnight. Following cooling to ambient temperature, saturated Na_2_SO_4_ (with water) was added to the mixture until no bubbles were observed. The solution was washed with diethyl ether (Et_2_O) several times, and the filtrate was dried over Na_2_SO_4_. The solvent was removed under reduced pressure, and the product was used without further purification.

#### (4-(Selenophen-2-yl)phenyl)methanamine^38^

Under an Ar atmosphere, 4-(selenophen-2-yl)benzonitrile (0.2 g, 0.86 mmol) was dissolved in dry THF (10 mL), and LiAlH_4_ (0.1 g, 2.6 mmol) was added. The reaction mixture was heated to 50 °C and stirred overnight. Then, the same procedure used to prepare (4-(thiophen-2-yl)phenyl)methanamine was followed.

#### (4-(Furan-2-yl)phenyl)methanamine^38^

The same procedure used to prepare (4-(thiophen-2-yl)phenyl)methanamine was followed, except with 4-(furan-2-yl)benzonitrile (0.3 g, 1.77 mmol) and LiAlH_4_ (0.25 g, 6.39 mmol).

#### (4-(Thiophen-2-yl)phenyl)methanaminium Iodide (TPMAI)^38^

At 0 °C, (4-(thiophen-2-yl)phenyl)methanamine (1.5 g, 7.9 mmol) was dissolved in ethanol (EtOH) (50 mL). Hydroiodic acid [HI (57 wt % in water)] (1.25 mL) was added slowly. The reaction mixture was allowed to stir for 1 h, and then, the solvent was removed. The precipitate was dissolved in EtOH and recrystallized by slow addition of Et_2_O. The resulting crystals were filtered and washed with cold Et_2_O several times and then dried under vacuum overnight. Yield: 1.5 g, 60%. ^1^H NMR (400 MHz, DMSO-*d*_6_): δ 8.13 (s, 3H), 7.73 (d, *J* = 8.2 Hz, 2H), 7.58 (t, *J* = 4.0 Hz, 2H), 7.49 (d, *J* = 8.0 Hz, 2H), 7.16 (dd, *J* = 5.0, 3.7 Hz, 1H), 4.06 (q, *J* = 5.6 Hz, 2H). ^13^C NMR (100 MHz, DMSO-*d*_6_): δ 142.9, 130.0, 128.9, 126.4,125.8, 124.5, 42.2.

#### (4-(Selenophen-2-yl)phenyl)methanaminium Iodide (SPMAI)^38^

Under an Ar atmosphere, (4-(selenophen-2-yl)phenyl)methanamine (0.55 g, 2.35 mmol) was dissolved in EtOH (mL). Following the addition of HI (57 wt % in water, 0.4 mL), the reaction mixture was allowed to stir for 1 h. Then, the same procedure used to prepare (4-(thiophen-2-yl)phenyl)methanaminium iodide was followed. Yield: 0.47 g, 62%. ^1^H NMR (400 MHz, DMSO-*d*_6_): δ 8.17 (d, *J* = 5.6 Hz, 1H), 8.13 (s, 3H), 7.74–7.65 (m, 3H), 7.47 (d, *J* = 8.0 Hz, 2H), 7.36 (dd, *J* = 5.6, 3.8 Hz, 1H), 4.06 (t, *J* = 5.4 Hz, 2H). ^13^C NMR (100 MHz, DMSO-*d*_6_): δ 131.8, 131.2, 130.0, 126.5, 126.3, 124.9, 42.2.

#### (4-(Furan-2-yl)phenyl)methanaminium Iodide (FPMAI)^38^

The same procedure used to prepare (4-(thiophen-2-yl)phenyl)methanaminium iodide was followed with (4-(furan-2-yl)phenyl)methanamine (0.24 g, 1.4 mmol) and HI (57 wt % in water, 0.25 mL). Yield: 0.25 g, 59%. ^1^H NMR (400 MHz, DMSO-*d*_6_): δ 8.13 (s, 3H), 7.76 (d, *J* = 7.7 Hz, 2H), 7.51 (d, *J* = 8.0 Hz, 2H), 7.01 (d, *J* = 3.4 Hz, 1H), 6.62 (p, *J* = 2.7 Hz, 2H), 4.06 (d, *J* = 5.5 Hz, 2H). ^13^C NMR (100 MHz, DMSO-*d*_6_): δ 152.8, 143.5, 133.2, 130.7, 129.8, 123.8, 112.5, 106.8, 42.3.

### Computational Details

The passivation of the perovskite surface by **FPMAI**, **TPMAI**, and **SPMAI** was investigated with density functional theory (DFT) using the Perdew–Burke–Ernzerhof (PBE)^39^ density functional along with D3 Grimme’s dispersion correction.^40^ The atomic basis set of localized Gaussian orbitals DZVP-MOLOPT^41^ was used to represent the wavefunction, while the charge density was expanded in the plane wave basis set with an energy cutoff of 600 Ry. The Γ-point was used to sample the reciprocal space. Norm-conserving Goedecker–Teter–Hutter^42^ (GHT) pseudopotentials were employed to describe the atomic core region and valence-core interactions. The calculations were performed with the CP2K code.^43^ Visualization was accomplished using VESTA software.^44^

The adsorption energies of the XPMAI salts with bulky organic cations are determined with the formula *E*_ads_(XPMAI-ML) = *E*_surf+xPMAI-ML_ – *E*_surf_ – *E*_xPMAI_, and the vacancy formation energies were calculated according to the formula Δ_f_*E*_I_vac_ = *E*_perovskite_I_vac_ + *E*_I_ – *E*_perovskite_.

### Fabrication of the PSCs

Fluorine-doped tin oxide (FTO)-coated substrates were washed with Hellmanex (2%), deionized water, acetone, and 2-propanol (IPA) for 10 min. The substrates were treated with UV-ozone for 15 min to eliminate organic residues on the surface. Then, titanium diisopropoxide bis(acetylacetonate) solution diluted in IPA at a 1:15 volume ratio to form a compact TiO_2_ (*c*-TiO_2_) film was coated by a spray pyrolysis method and annealed at 450 °C for 30 min. Mesoporous TiO_2_ (*mp*-TiO_2_) solution was prepared by dissolving 1 g of TiO_2_ paste in ethanol at a volume ratio of 1:10 and was coated on the compact TiO_2_ layer by rotating at 4500 rpm for 20 s. Samples were first annealed at 125 °C for 30 min, with the temperature gradually increased to 525 °C, and annealed at this temperature for another 20 min. The SnO_2_ layer was spin-coated using the 0.1 M SnCl_4_ solution at 3000 rpm for 20 s, annealed at 150 °C for 10 min, and then at 190 °C for 1 h. Before coating the perovskite solution, the precoated film was treated with UV-ozone for 30 min. The perovskite precursor solution was prepared by dissolving the lead iodide (PbI_2_) (1.19 M), formamidinium iodide (FAI) (1.04 M), methylammonium bromide (MABr) (0.15 M), cesium iodide (CsI) (0.10 M), and lead bromide (PbBr_2_) (0.15 M) in *N*,*N*-dimethylformamide:dimethyl sulfoxide (DMF:DMSO) (4:1 volume ratio). The prepared solution was first coated at 1000 rpm for 10 s and then at 5000 rpm for 30 s, and 100 μL of chlorobenzene was dropped onto the film surface 15 s before the end of the coating process. The film was annealed at 100 °C for 1 h. Then, 10 mg amounts of **TPMAI**, **SPMAI**, and **FPMAI** salts were dissolved in 1 mL of IPA. The salt solutions were spin-coated at 4000 rpm for 30 s. Spiro-OMeTAD:Co (FK209):Li-TFSI:TBP was mixed at molar ratios of 1:0.03:0.5:3.3 and spin-coated at 4000 rpm for 20 s. Finally, a 70 nm-thick gold (Au) layer was deposited as the counter electrode by evaporation. The active area of the device was 0.09 cm^2^.

## Supporting Information Available

UPS spectra of reference and XPMAI-treated films; full photovoltaic parameters of the devices; TRPL fitting parameters; contact angle images of the reference, FPMAI, TPMAI, and SPMAI-treated films; NMR spectra for all compounds (PDF)
