# Supplementary material for: Functionalized Phenyl Methanaminium Salts Provide Highly Stable Perovskite Solar Cells
Source: ACS Appl Mater Interfaces. 2025 Mar 17;17(12):18450–7. doi: 10.1021/acsami.5c00985 (PMC11956009; doi:10.1021/acsami.5c00985)
Supplement: Supplementary file 1 — am5c00985_si_001.pdf [file am5c00985_si_001.pdf]

Supporting Information

Functionalized Phenyl-methanaminium Salts Provide Highly Stable Perovskite Solar Cells

Mustafa Yaşa,[1,2] Esra Bag Celik,[1,2] Xiao-Xin Gao,[3] Zeynep Gözükara Karabağ,[1,3,4] Ummugulsum Gunes,[1,3] Olga A. Syzgantseva,[5] Maria A. Syzgantseva,[6] Liping Zhong,[3] Andreas Züttel,[3] Paul Dyson,[3*] Mohammad Khaja Nazeeruddin[3*], Levent Toppare[2,4], Selcuk Yerci,[1,7,8] Gorkem Gunbas[1,2,4,7*]*

[1]*ODTÜ-GÜNAM, Middle East Technical University, Ankara 06800, Turkey*

[2]*Department of Polymer Science and Technology, Middle East Technical University, Ankara 06800, Turkey*

[3]*Institute of Chemical Sciences and Engineering, École Polytechnique Fédérale de Lausanne, 1015 Lausanne, Switzerland*

[4]*Department of Chemistry, Middle East Technical University, Ankara 06800, Turkey*

[5]*Department of Chemistry, Lomonosov Moscow State University, 119991 Moscow, Russia*

[6]*Department of Physics, Mendeleev University of Chemical Technology, 125047, Moscow, Russia*

[7]*Department of Micro and Nanotechnology, Middle East Technical University, Ankara 06800, Turkey*

[8]*Department of Electrical-Electronic Engineering, Middle East Technical University, Ankara 06800, Turkey*

xiaoxin.gao@epfl.ch, paul.dyson@epfl.ch, mdkhaja.nazeeruddin@epfl.ch, ggunbas@metu.edu.tr

UPS Spectra

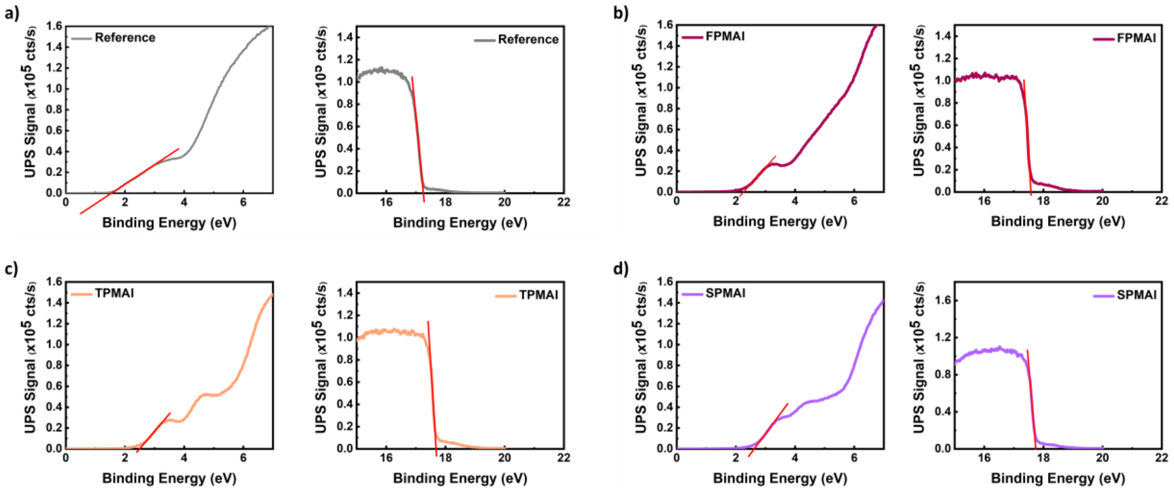

Figure S1. UPS spectra of reference and PMAI-treated films. The valence band maxima cut-off regions and work function regions of **a)** reference, **b) FPMAI**, **c) TPMAI**, and **d) SPMAI**-treated films.

Table S1. Reverse and forward measurement *J–V* parameters of champion reference, **FPMAI**, **TPMAI** and **SPMAI** PSCs.

Device	V_{OC} (V)	J_{SC} (mA/cm^2)	FF (%)	PCE %	HI (%)
Reference – reverse	1.08	24.98	77.5	20.91	7.75
Reference – forward	1.09	24.83	71.4	19.29	
FPMAI – reverse	1.12	25.13	78.7	22.18	4.78
FPMAI – forward	1.11	25.04	76.0	21.12	
TPMAI – reverse	1.13	25..67	80.7	23.15	4.69
TPMAI – forward	1.11	25.66	78.5	22.37	
SPMAI – reverse	1.12	24.73	78.5	21.65	7.20
SPMAI – forward	1.10	24.87	73.4	20.09	

Time-resolved Photo Luminescence (TRPL)

A typical bi-exponential function was applied for TRPL decay fitting [1]. **Table S2** shows the fitting parameters of TRPL spectra.

Table S2. Fitting parameters of TRPL spectra.

Device	A_1	τ_1	A_2	τ_2	R^2
Reference	0.312	235.1	0.567	1080.9	0.991
FPMAI	0.242	415.1	0.587	1546.1	0.982
TPMAI	0.315	506.8	0.454	1790.9	0.963
SPMAI	0.376	266.9	0.489	1250.9	0.984

Contact Angle

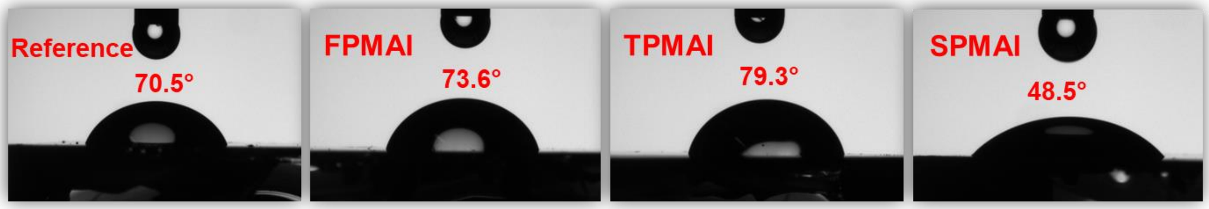

Figure S2. Contact angle images of the reference, **FPMAI**, **TPMAI** and **SPMAI** films.

NMR Spectra

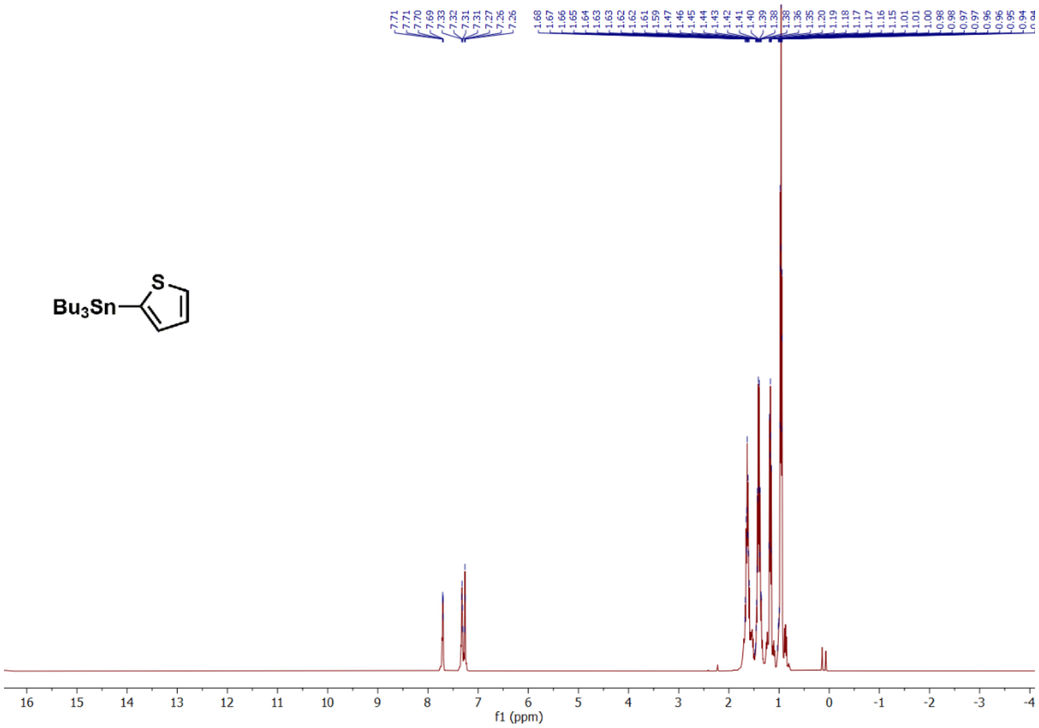

Figure S3. ^{1}H NMR spectrum of tributyl(thiophen-2-yl)stannane in $CDCl_3$.

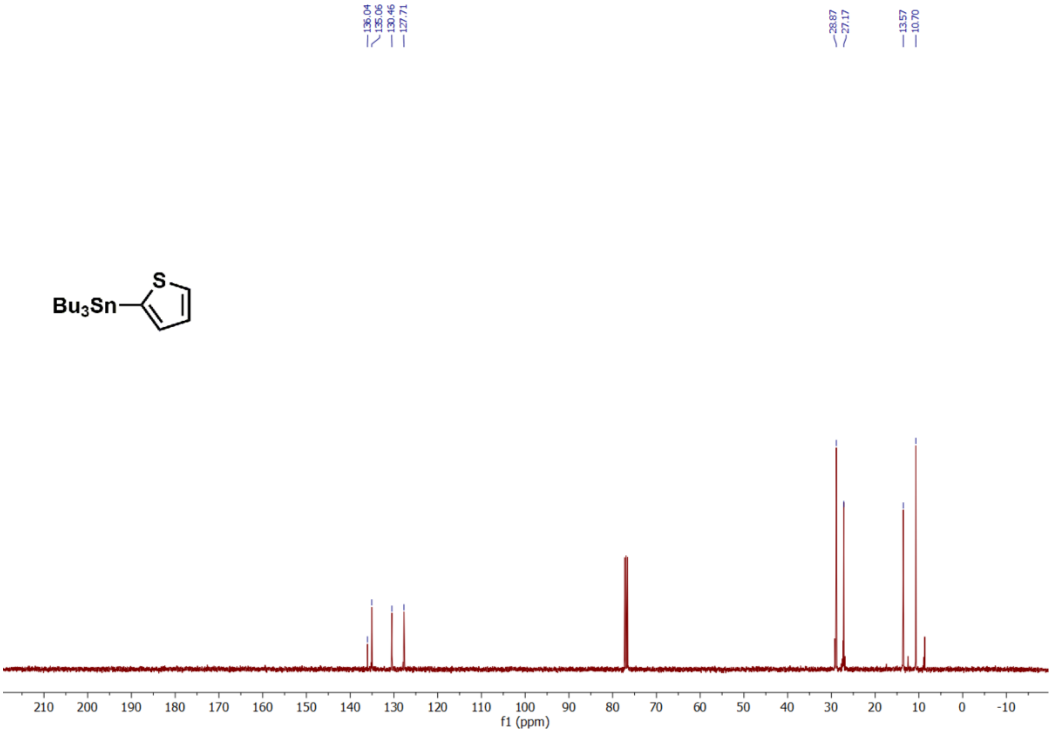

Figure S4. ^{13}C NMR spectrum of tributyl(thiophen-2-yl)stannane in $CDCl_3$.

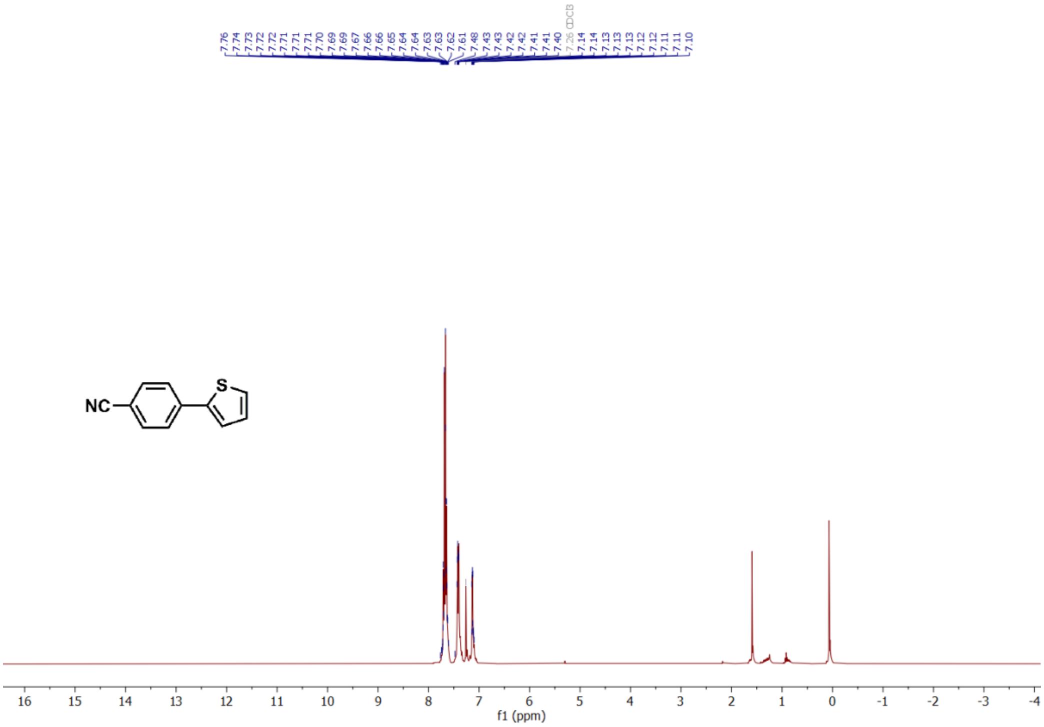

Figure S5. ^{1}H NMR spectrum of 4-(thiophen-2-yl)benzonitrile in $CDCl_3$.

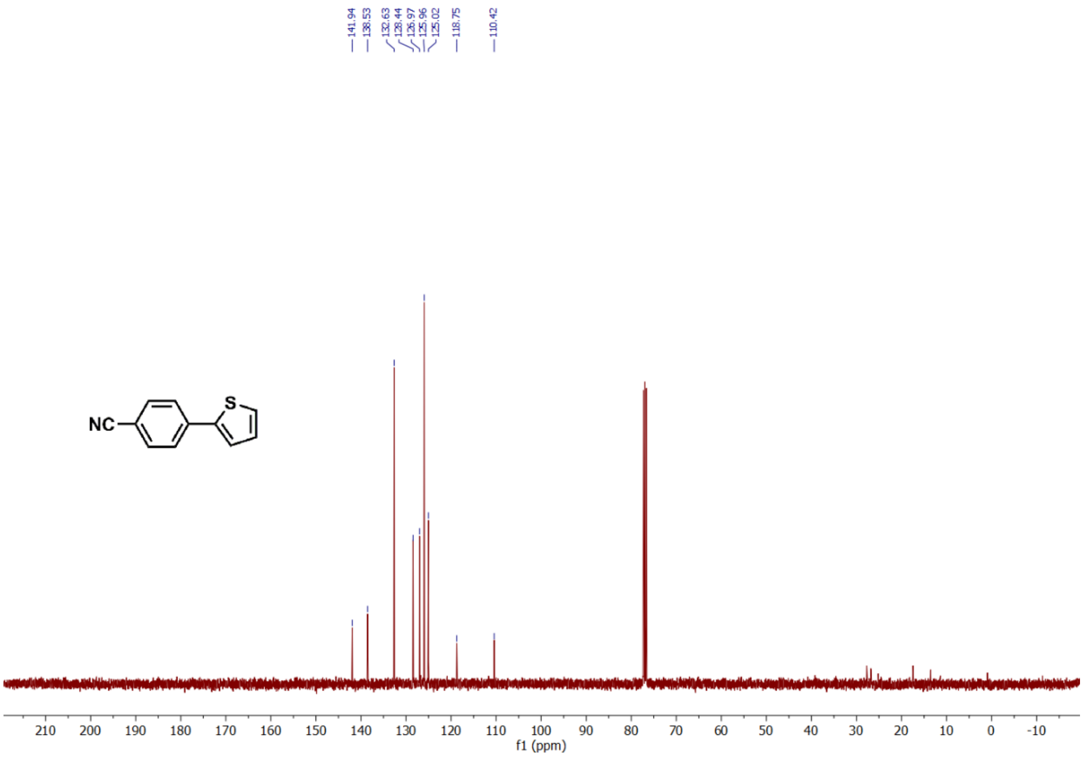

Figure S6. ^{13}C NMR spectrum of 4-(thiophen-2-yl)benzonitrile in $CDCl_3$.

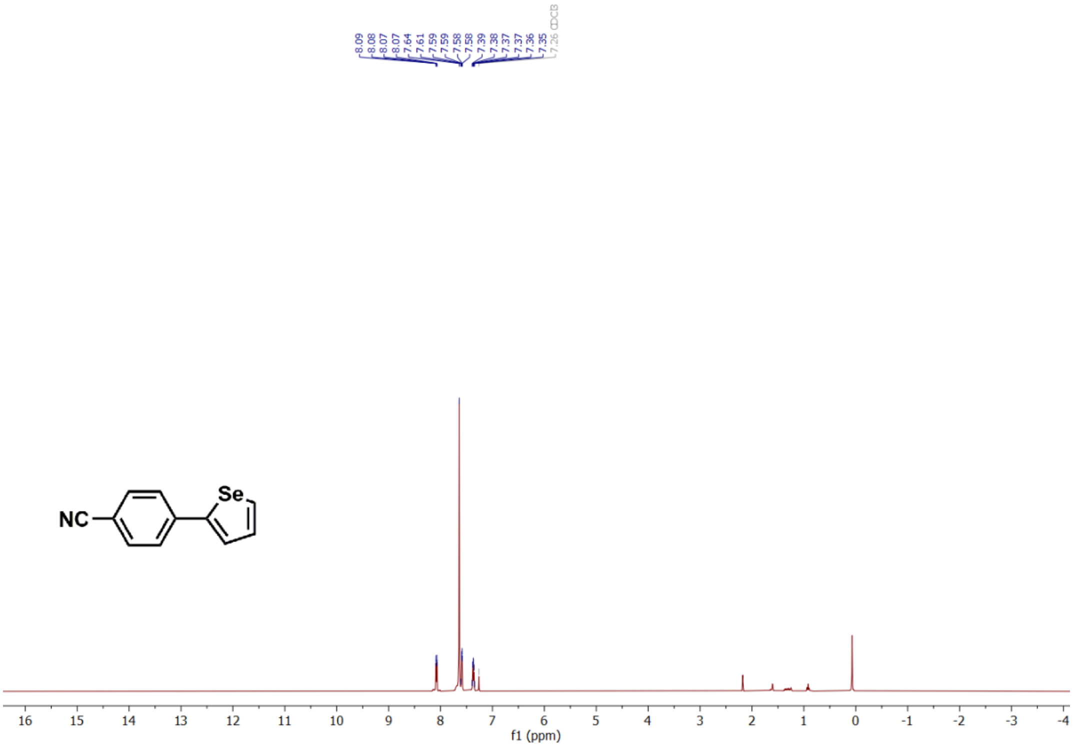

Figure S7. ^{1}H NMR spectrum of 4-(selenophen-2-yl)benzonitrile in $CDCl_3$.

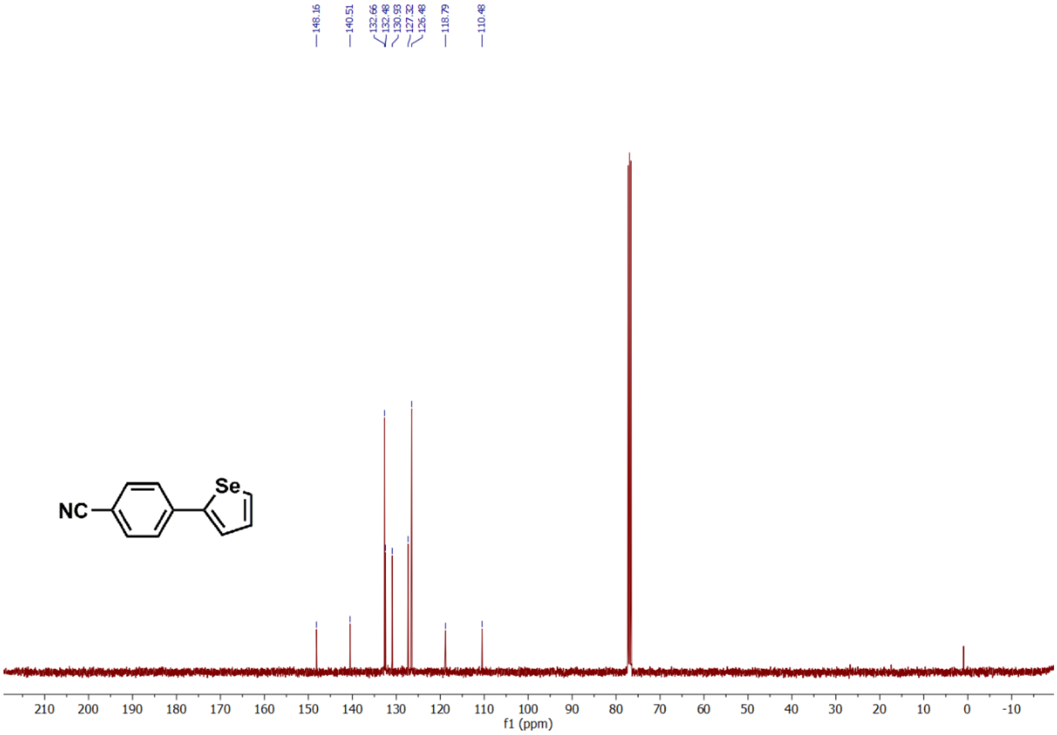

Figure S8. ^{13}C NMR spectrum of 4-(selenophen-2-yl)benzonitrile in $CDCl_3$.

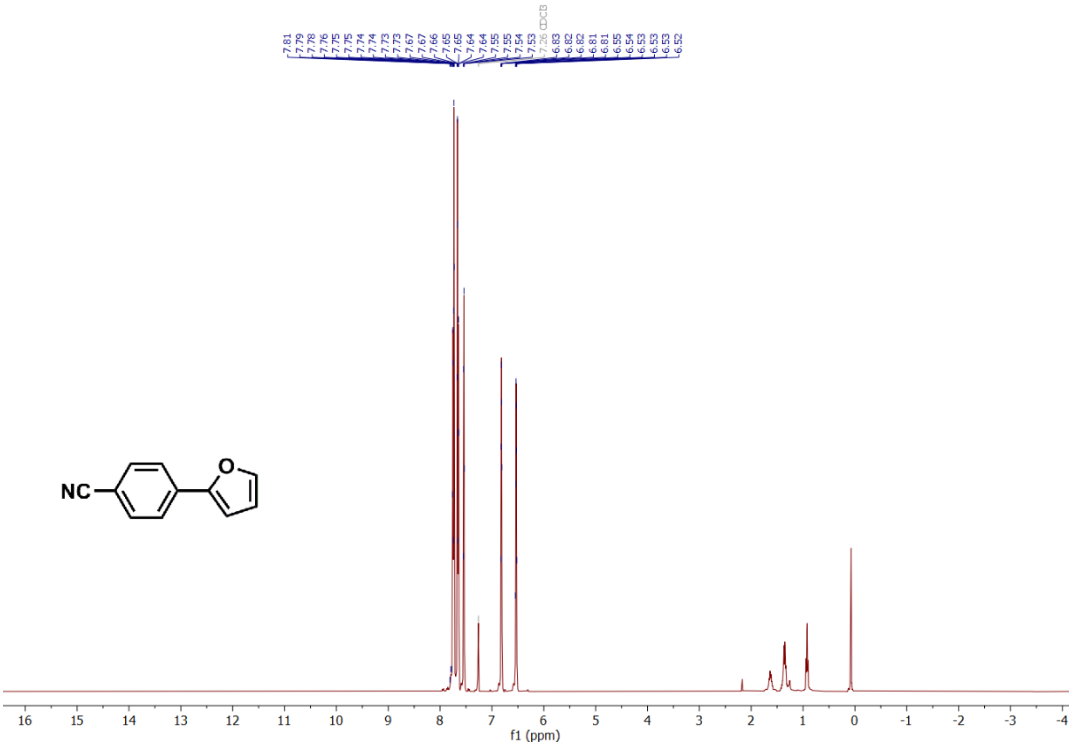

Figure S9. ^{1}H NMR spectrum of 4-(furan-2-yl)benzonitrile in $CDCl_3$.

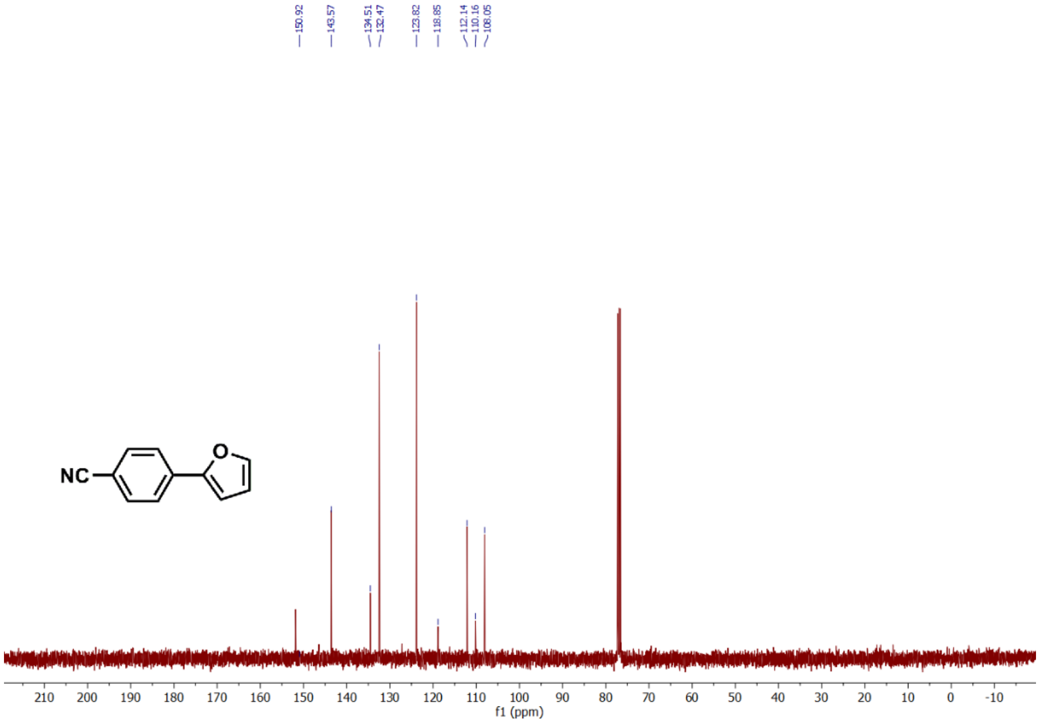

Figure S10. ^{13}C NMR spectrum of 4-(furan-2-yl)benzonitrile in $CDCl_3$.

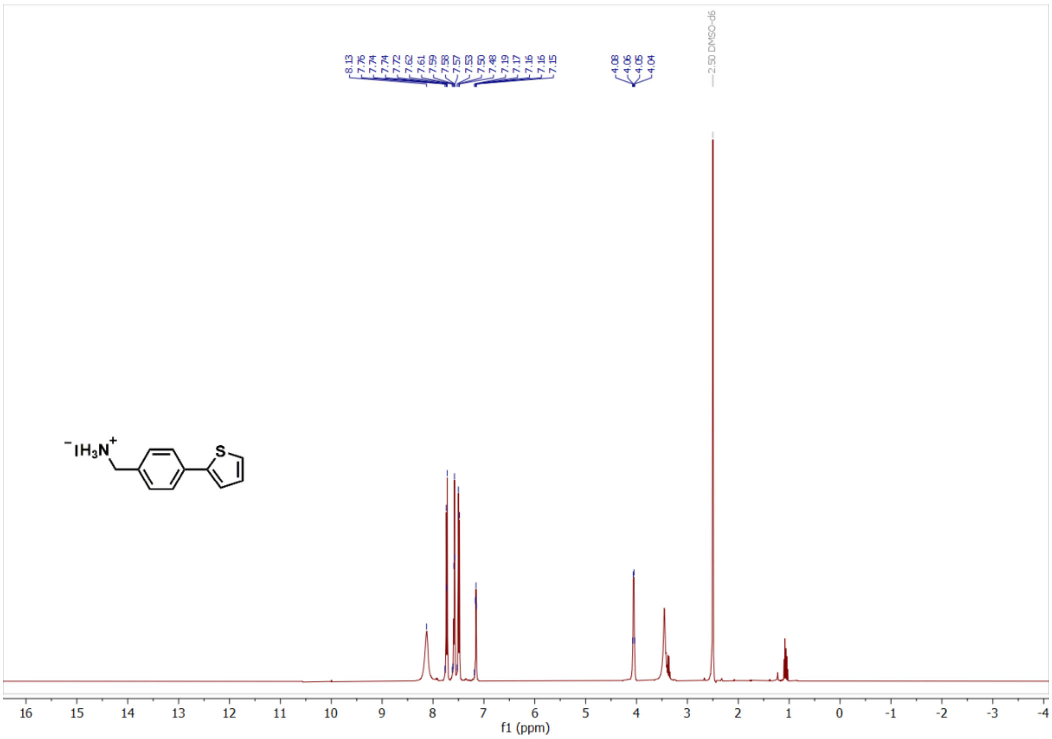

Figure S11. ^{1}H NMR spectrum of **TPMAI** in DMSO-d_6.

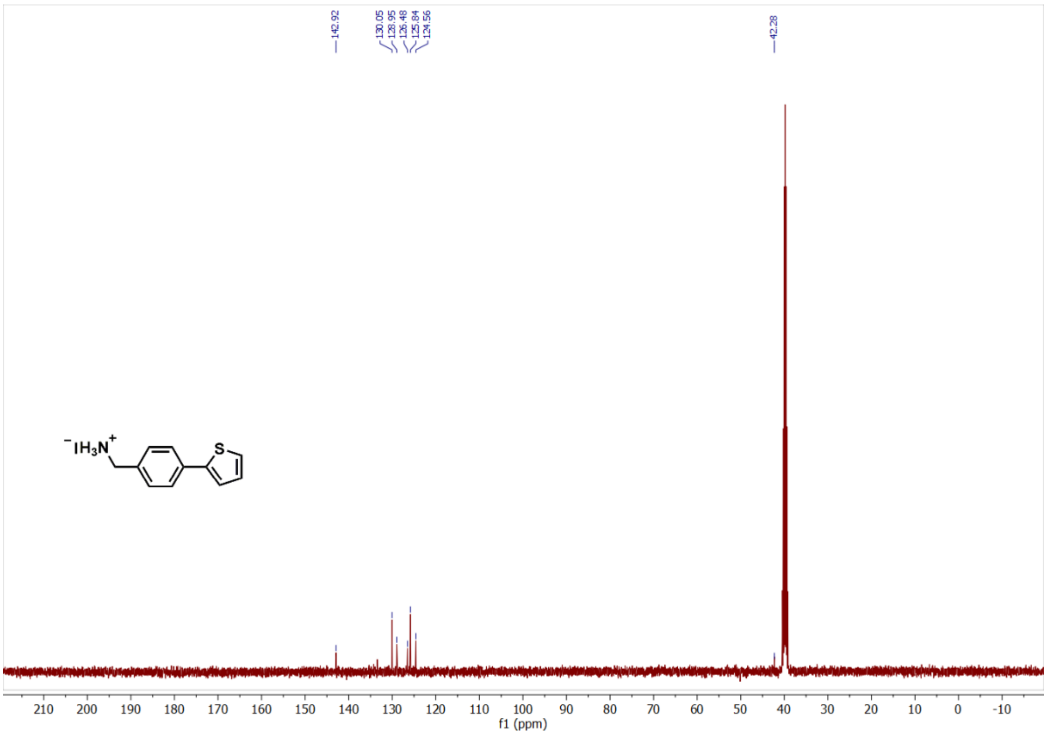

Figure S12. ^{13}C NMR spectrum of **TPMAI** in DMSO-d_6.

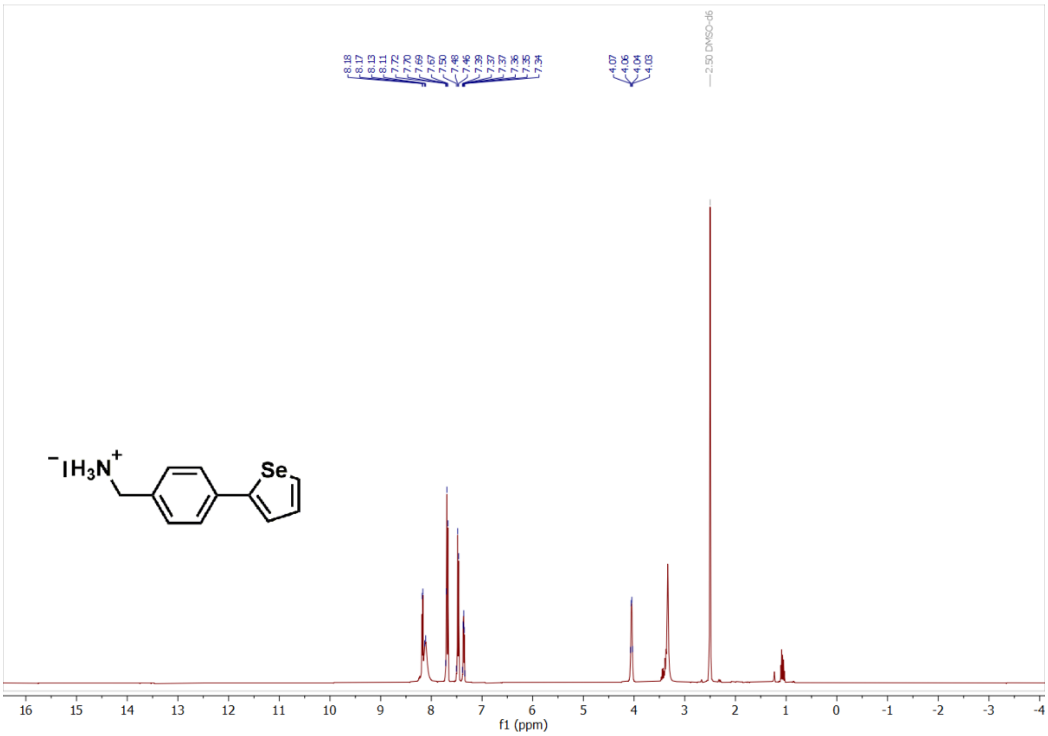

Figure S13. ^{1}H NMR spectrum of **SPMAI** in DMSO-d$_6$.

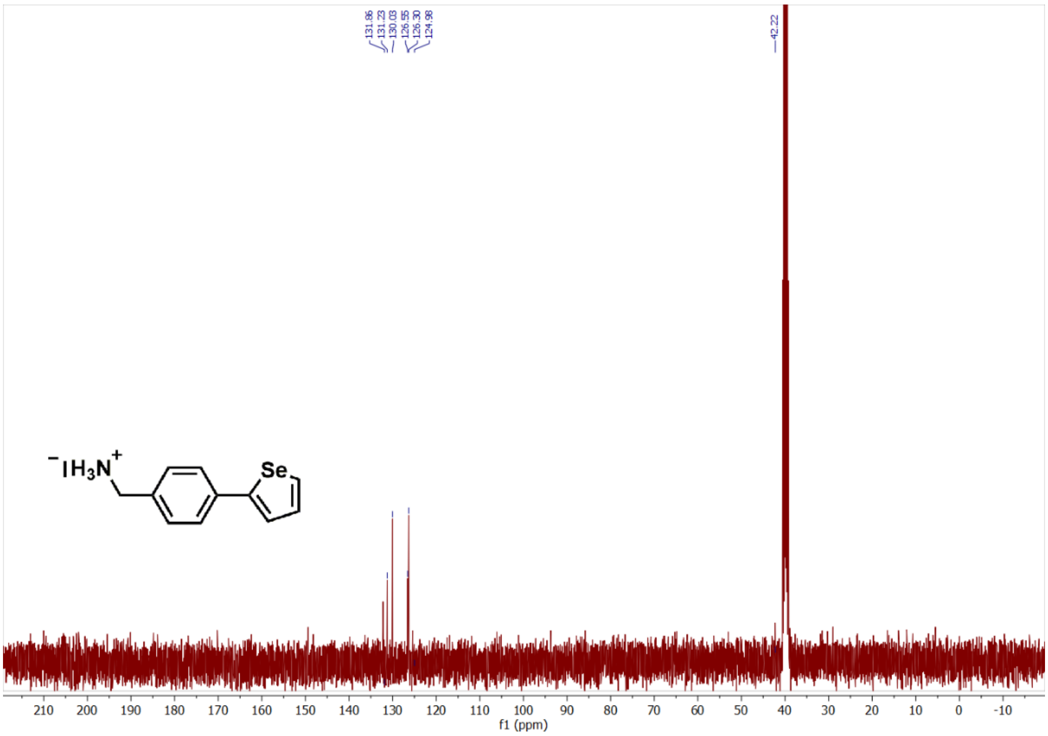

Figure S14. ^{13}C NMR spectrum of **SPMAI** in DMSO-d$_6$.

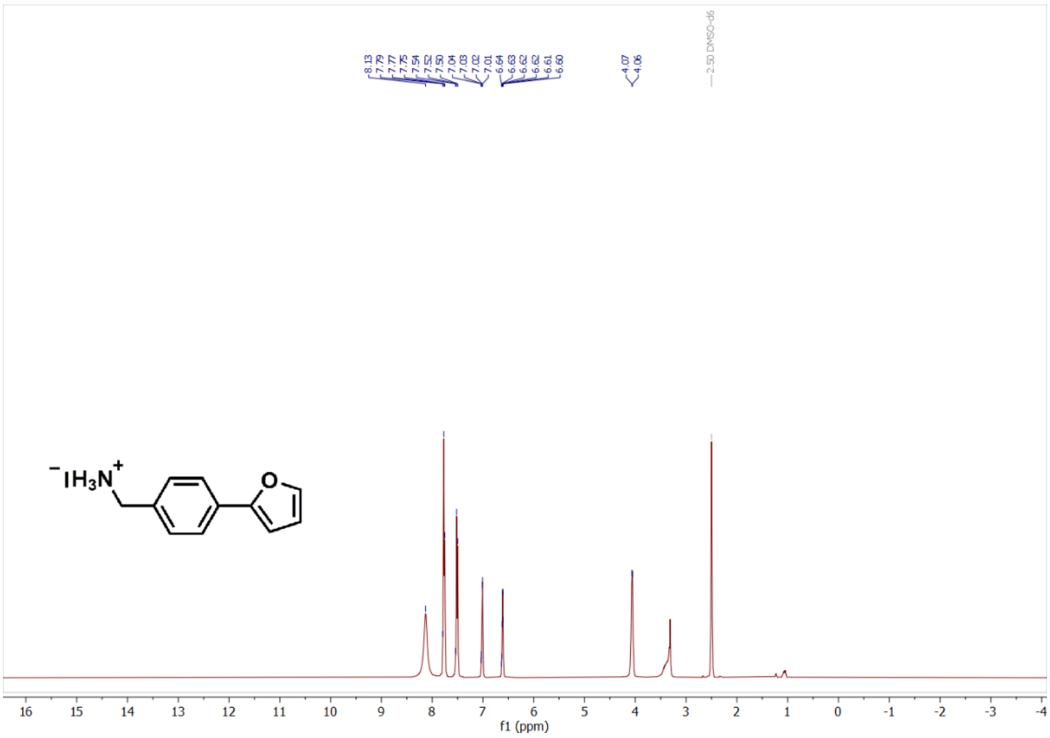

Figure S15. ^{1}H NMR spectrum of **FPMAI** in DMSO-d_6.

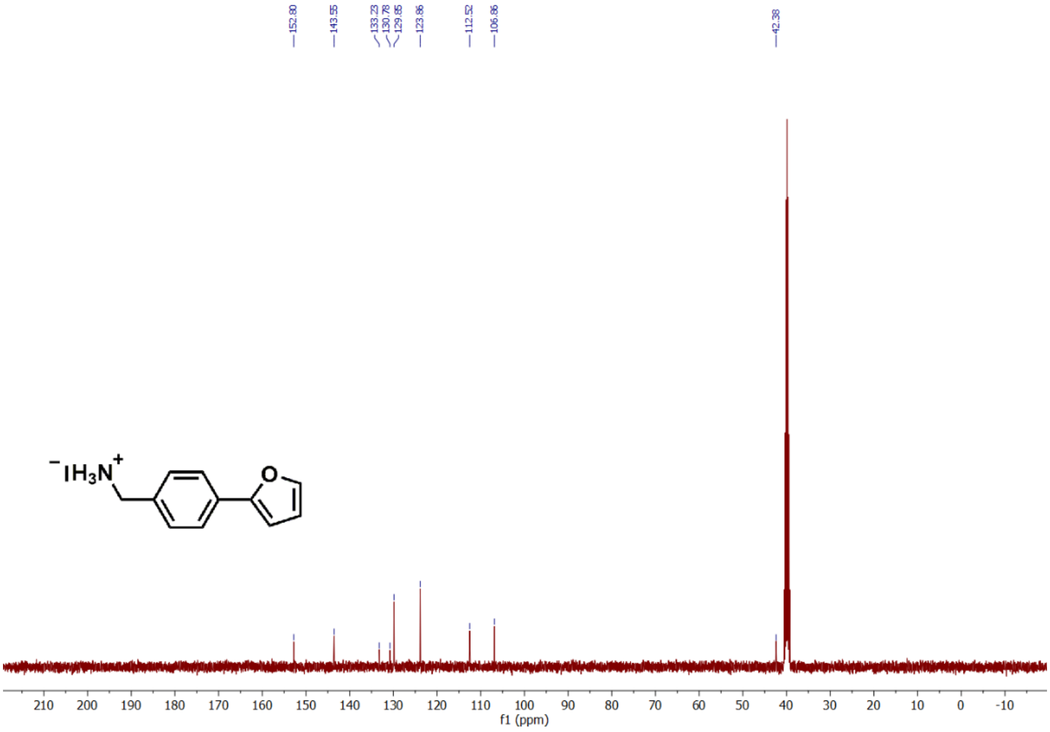

Figure S16. ^{13}C NMR spectrum of **FPMAI** in DMSO-d_6.

REFERENCES

(S1) Péan, E. V.; Dimitrov, S.; De Castro, C. S.; Davies, M. L.Interpreting Time-Resolved Photoluminescence of Perovskite Materials. *Phys. Chem. Chem. Phys.* 2020, 22 (48), 28345−28358. https://doi.org/10.1039/D0CP04950F.
